# Right-side versus left-side hepatectomy for the treatment of hilar cholangiocarcinoma: a comparative study

**DOI:** 10.1186/s12957-019-1779-1

**Published:** 2020-01-04

**Authors:** Hye-Sung Jo, Dong-Sik Kim, Young-Dong Yu, Woo-Hyoung Kang, Kyung Chul Yoon

**Affiliations:** Division of HBP Surgery and Liver Transplantation, Department of Surgery, Korea University College of Medicine, 73, Goryedae-ro, Seongbuk-gu, Seoul, 02841 Korea

**Keywords:** Hilar cholangiocarcinoma, Hepatectomy, Radical resection

## Abstract

**Background:**

Radical resection is the only curative treatment for patients with hilar cholangiocarcinoma. While left-side hepatectomy (LH) may have an oncological disadvantage over right-side hepatectomy (RH) owing to the contiguous anatomical relationship between right hepatic inflow and biliary confluence, a small future liver remnant after RH could cause worse surgical morbidity and mortality. We retrospectively compared surgical morbidity and long-term outcome between RH and LH to determine the optimal surgical strategy for the treatment of hilar cholangiocarcinoma.

**Methods:**

This study considered 83 patients who underwent surgical resection for hilar cholangiocarcinoma between 2010 and 2017. Among them, 57 patients undergoing curative-intent surgery including liver resection were enrolled for analysis—33 in the RH group and 27 in the LH group. Prospectively collected clinicopathologic characteristics, perioperative outcomes, and long-term survival were evaluated.

**Results:**

Portal vein embolization was more frequently performed in the RH group than in the LH group (18.2% vs. 0%, *P =* 0.034). The proportion of R0 resection was comparable in both groups (75.8% vs. 75.0%, *P =* 0.948). The 5-year overall and recurrence-free survival rates did not differ between the groups (37.7% vs. 41.9%, *P =* 0.500, and 26.3% vs. 33.9%, *P =* 0.580, respectively). The side of liver resection did not affect long-term survival. In multivariate analysis, transfusion (odds ratio, 3.12 [1.42–6.87], *P =* 0.005) and post-hepatectomy liver failure (≥ grade B, 4.62 [1.86–11.49], *P =* 0.001) were independent risk factors for overall survival.

**Conclusions:**

We recommend deciding the side of liver resection according to the possibility of achieving radical resection considering the anatomical differences between RH and LH.

## Background

Complete surgical resection with a negative margin is the only curative treatment for hilar cholangiocarcinoma [1–3]. However, R0 resection is always technically demanding owing to the complex contiguity of the hilar structures and longitudinal spread of the tumor. Surgical morbidity and mortality are relatively high since surgical resection for hilar cholangiocarcinoma usually consists of extensive resection including major hepatectomy [4, 5].

Regarding the extent of liver resection, inclusion of the caudate lobe has been a standard procedure as the bile ducts of the caudate lobe originate in the hilar bile ducts [6, 7]. Right-side or left-side hepatectomy (RH or LH, respectively) is also mandatory to achieve a negative margin for hilar cholangiocarcinoma above Bismuth type II [1, 8]. Which side of the liver to resect is determined according to the following considerations: (1) side and level of intrahepatic bile duct invaded by the tumor, (2) vascular invasion to the hepatic artery or portal vein, and (3) adequate future liver remnant (FLR) volume.

Tumors often invade the right hepatic artery because the right hepatic artery usually courses close behind the biliary confluence. When performing LH in such cases, aggressive vascular reconstruction is required to achieve radical resection [9, 10]. Hence, some have argued that LH is considered to have an oncological disadvantage over RH [11]. However, a small FLR after RH could lead to post-hepatectomy liver failure (PHLF) and relatively high morbidity and mortality [12]. There have been few studies about the comparative analysis between RH and LH in hilar cholangiocarcinoma, and the impact of the side of liver resection has not yet been fully determined [12, 13].

Therefore, the aim of this study was to compare surgical morbidity and long-term outcomes between RH and LH in patients undergoing curative-intent resection for hilar cholangiocarcinoma.

## Methods

### Patients

**Fig. 1:**
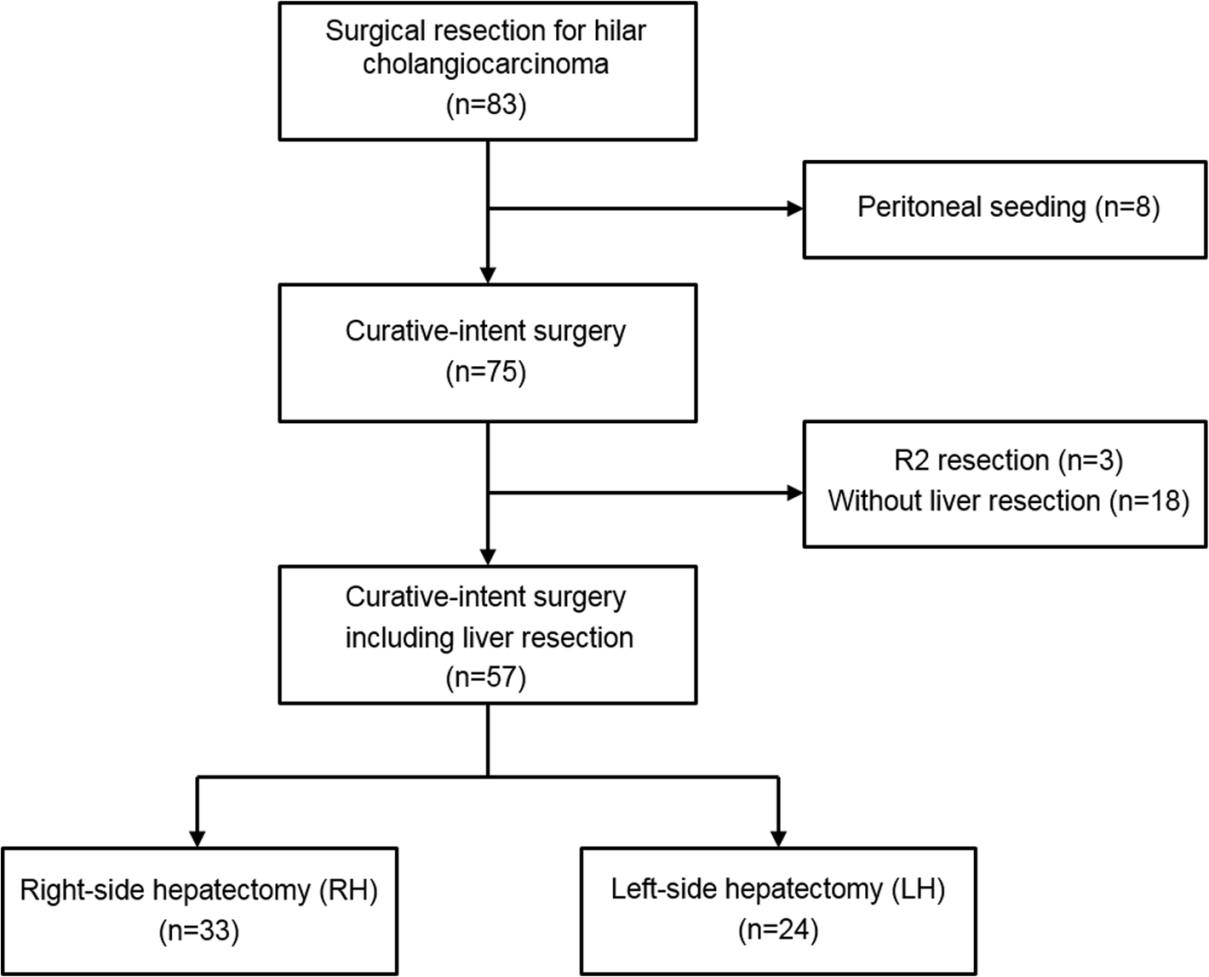
Patient flow chart depicting the cohort selection

All 83 consecutive patients who underwent surgical resection for hilar cholangiocarcinoma between 2010 and 2017 were considered for this study. The following exclusion criteria were applied: (1) non-curative-intent surgery such as bypass surgery, (2) surgery without liver resection, and (3) R2 resection (macroscopic residual tumor). The resulting study cohort comprised 57 patients: 33 in the RH group and 27 in the LH group (Fig. 1). Prospectively collected data were retrospectively reviewed. This study was approved by the Institutional Review Board of Korea University Anam Hospital (2019AN0411).

### Preoperative evaluation

Contrast-enhanced multidetector computed tomography (CT) and magnetic resonance (MR) cholangiography were routinely performed to assess the tumor extent and resectability as well as anatomical variation. In addition, positron emission tomography (PET)-CT was performed to rule out potential distant metastases.

In patients with obstructive jaundice, preoperative biliary drainage, consisting of endoscopic nasobiliary drainage (ENBD), through endoscopic retrograde cholangiopancreatography (ERCP) or percutaneous transhepatic biliary drainage (PTBD) were aggressively performed not only to obtain a histological diagnosis but also to decrease the bilirubin level. The indocyanine green (ICG) test was performed to assess the functional status of the liver after the total bilirubin level decreased to below 2.0 mg/dL. Achievement of radical resection was the most important consideration in determining the surgical strategy. The longitudinal and radial extent of the tumor was assessed comprehensively through various imaging studies during an inter-department conference. Preoperative portal vein embolization (PVE) was considered for patients who had planned RH if the FLR volume was less than 35% of the total liver volume, as assessed by CT volumetry.

### Surgical procedure

The surgical procedures are detailed as follows. After making an upper midline incision, the entire abdominal cavity was explored to detect unexpected peritoneal seeding or metastasis. If there was no obvious distant metastasis, a transverse extension to the right side was made to just below the right subcostal margin. A Kocher maneuver was routinely performed for resection of the aortocaval and retropancreatic lymph nodes. The hepatic artery and portal vein to the FLR were isolated to evaluate tumor resectability. Thereafter, the distal common bile duct was isolated and divided at the level of the intrapancreatic portion to retain a negative distal resection margin, and the remainder was sent for frozen biopsy. Subsequently, skeletonization of the hepatoduodenal ligament was performed. The hepatic artery and portal vein of the side to be resected were suture-tied and divided, being careful not to disturb the vascular inflow to the FLR. If the tumor invaded the portal vein confluence, segmental resection and anastomosis were performed before liver transection. After the liver was mobilized by dividing all ligamentous attachments, it was transected using a Cavitron Ultrasonic Surgical Aspirator (Valleylab, Boulder, Colorado, USA) along the demarcation line marked by the ischemic color change of the liver surface. The caudate lobe was involved in all cases. The left intrahepatic bile duct was resected at the origin of the umbilical portion in RH, while the right intrahepatic bile duct was resected at the highest achievable level in LH. The hepatic vein was then resected.

Multiple bile duct openings usually remained to be reconstructed after removal of the specimen from the abdominal cavity. The Roux limb was placed up in a retrocolic fashion, and hepaticojejunostomy was performed using a single-layer suture after making the bile duct openings contiguous whenever possible. After completing the posterior wall suture, a trans-anastomotic internal plastic stent was inserted into each opening. Thereafter, jejunojejunostomy was performed. Two drainage catheters were placed around the resection plane of the liver and the hepaticojejunostomy. Abdominal closure was performed after hemostasis was achieved.

### Postoperative follow-up

Patients underwent clinical follow-up every 3 months for the first year and every 6 months thereafter. The follow-up visits comprised a physical exam, laboratory tests including tumor markers, and CT scan. Postoperative adjuvant treatment was performed based on the final pathologic report. All patients who had lymph node metastasis were attempted to receive adjuvant chemotherapy with gemcitabine plus cisplatin, except for patients who refused. Patients with positive resection margin (R1 resection) received 5-FU-based concurrent chemoradiotherapy. No postoperative treatment was performed for patients who had no lymph node metastasis after R0 resection.

### Definition

The Bismuth–Corlette classification was used to categorize the type of hilar cholangiocarcinoma, as assessed by various imaging studies [14]. Preoperative cholangitis was defined as fever with increased bilirubin and white blood cell count with antibiotic administration. PHLF was defined according to the International Study Group of Liver Surgery [15], and complications were graded according to Clavien–Dindo classifications [16]. T and N staging was based on the American Joint Committee on Cancer 7^th^ edition.

### Statistical analysis

Continuous variables are presented as median and range and categorical variables as numbers with percentages. Comparison of continuous variables between groups was performed using Student’s *t* and Mann–Whitney *U* tests. Categorical variables were compared using *χ*^2^ or Fisher’s exact tests, as appropriate. Overall and recurrence-free survival (OS and RFS, respectively) were calculated using Kaplan–Meier analysis and compared using log-rank tests. Cox proportional hazards regression analysis was used to assess the prognostic significance of variables for survival. Multivariate analysis was performed on factors with *P* values ≤ 0.1 by univariate analysis. *P* values < 0.05 denoted statistical significance. IBM SPSS Statistics for Windows version 20.0 was used for all statistical analyses (IBM Corp., Armonk, NY, USA).

## Results

### Baseline characteristics

Baseline characteristics for all patients are shown in Table 1. These included 37 male and 20 female patients, with a median age of 66 (42–83) years. The median follow-up was 19 (1–97) months. Only one patient had an underlying hepatitis B virus infection in the LH group (0 % vs. 4.2%, *P* = 0.421), and no patient in both groups had hepatitis C virus infection. Among patients who received preoperative biliary drainage, ENBD was performed for 20 patients (68.9%) in the RH group and 14 patients (63.6%) in the LH group (*P =* 0.856); the remaining patients underwent PTBD. Initial total bilirubin upon hospital referral was higher in the RH group than that in the LH group, with borderline significance (5.36 [0.35–24.96] vs. 1.51 [0.48–21.88], *P =* 0.093). However, there was no difference in total bilirubin prior to surgery (1.30 [0.37–3.47] vs. 0.90 [0.47–2.76], *P =* 0.281) and the duration jaundice relief between groups (18 [3–49] days vs. 11 [6–29] days, *P =* 0.218). Six patients in the RH group underwent portal vein embolization because of a small FLR volume, compared to no patients in the LH group (18.2% vs. 0%, *P =* 0.034). The baseline characteristics, besides portal vein embolization, did not differ between groups.

**Table 1:** Baseline characteristics

	RH group (*n* = 33)	LH group (*n* = 24)	*P* value
Clinical variables			
Age	66 (42–79)	71 (53–83)	0.047
Sex (male)	22 (66.6%)	15 (62.5%)	0.745
BMI (kg/m^2^)	23.2 (18.2–33.0)	23.8 (20.2–34.9)	0.702
Bismuth type IV	12 (36.4%)	7 (29.2%)	0.587
Preoperative cholangitis	14 (42.4%)	8 (33.3%)	0.486
Preoperative biliary drainage	29 (87.9%)	22 (91.7%)	1.000
Portal vein embolization	6 (18.2%)	0 (0%)	0.034
Total bilirubin prior to surgery (mg/dL)	1.30 (0.37–3.47)	0.90 (0.47–2.76)	0.281
ICG R-15 (%)	12.6 (3.0–19.7)	12.5 (4.7–26.7)	0.816
CEA (ng/mL)	1.4 (0.2–12.1)	2.1 (0.5–33.1)	0.198
CA 19-9 (IU/mL)	86.8 (7.9–7514.0)	77.3 (1.1–10404.0)	0.456
Operative variables			
Operation time (min)	557 (415–975)	525 (240–1127)	0.189
Portal vein resection	9 (27.3%)	2 (8.3%)	0.097
Transfusion	13 (39.4%)	9 (37.5%)	0.885
PHLF (≥ grade B)	7 (21.2%)	2 (8.3%)	0.277
Complication (≥ IIIA)	14 (42.4%)	10 (41.7%)	0.954
Hospital stay (days)	20 (12–239)	19 (8–171)	0.862
90-day mortality	3 (9.1%)	1 (4.2%)	0.631
Pathologic variables			
R0 resection	25 (75.8%)	18 (75.0%)	0.948
Tumor size (cm)	3.5 (1.2–8.5)	3.8 (0.8–7.0)	0.733
Differentiation (poorly)	4 (13.8%)	2 (11.8%)	1.000
Lymphovascular invasion	15 (45.5%)	14 (58.3%)	0.337
Perineural invasion	25 (75.8%)	19 (79.2%)	0.762
T stage (≥ T3)	9 (27.3%)	7 (29.2%)	0.875
N stage (≥ N1)	15 (45.5%)	6 (25.0%)	0.114

*Abbreviations: RH* right-side hepatectomy, *LH* left-side hepatectomy, *BMI* body mass index, *ICG R-15* indocyanine green retention rate at 15 min, *PHLF* post-hepatectomy liver failure

Values are presented as median (range) for continuous data and n (%) for categorical data

Subgroup analyses of the RH group showed no differences in the occurrence of PHLF (≥ grade B) and postoperative complications (≥ grade IIIA) between the PVE and non-PVE groups (16.7% vs. 18.5%, *P =* 0.705, and 33.3% vs. 44.4%, *P =* 1.000, respectively). In terms of mortality, none of the patients in the PVE group died before postoperative day 90, compared to three patients in the non-PVE group (0% vs. 11.1%, *P =* 0.614).

### Ninety-day mortality

In this study, four patients died within 90 days after surgery (7.0%), including three and one patient in the RH and LH group (9.1% vs. 4.2%, *P =* 0.631), respectively. One patient in the RH group died due to grade C PHLF. Although the FLR was over 35% and preoperative liver function was preserved, the total bilirubin and ammonia levels gradually increased after surgery. With a combined intra-abdominal infection, hepatic failure progressed and the patient died at postoperative day 25. Two patients in the RH group died due to pneumonia-induced sepsis. One patient in the LH group developed a pseudoaneurysm of the hepatic artery after biliary leakage for which a stent graft was inserted successfully. However, liver abscess and pneumonia-induced sepsis occurred subsequently.

### Survival analyses

**Fig. 2:**
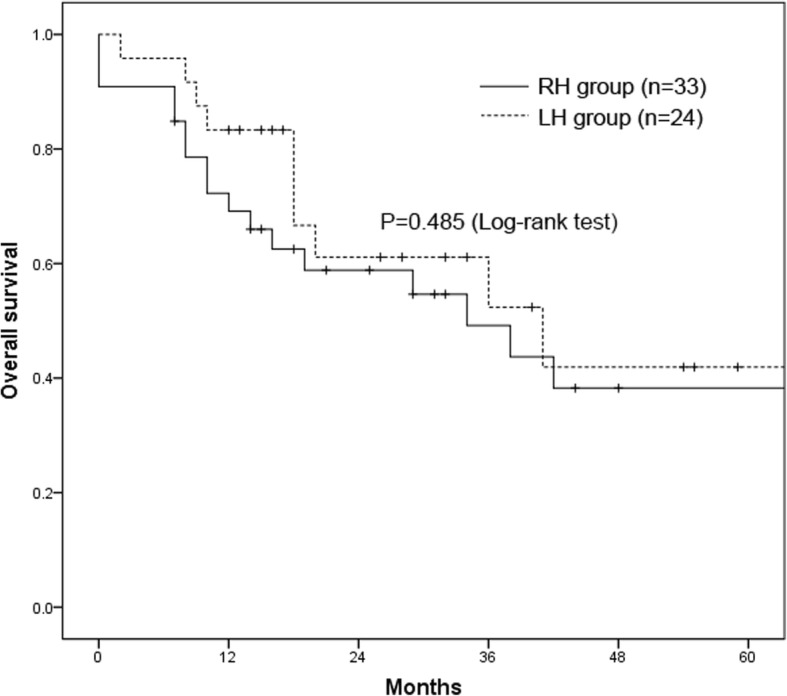
Kaplan–Meier curves showing overall survival between the RH and LH groups. RH, right-side hepatectomy; LH, left-side hepatectomy

**Fig. 3:**
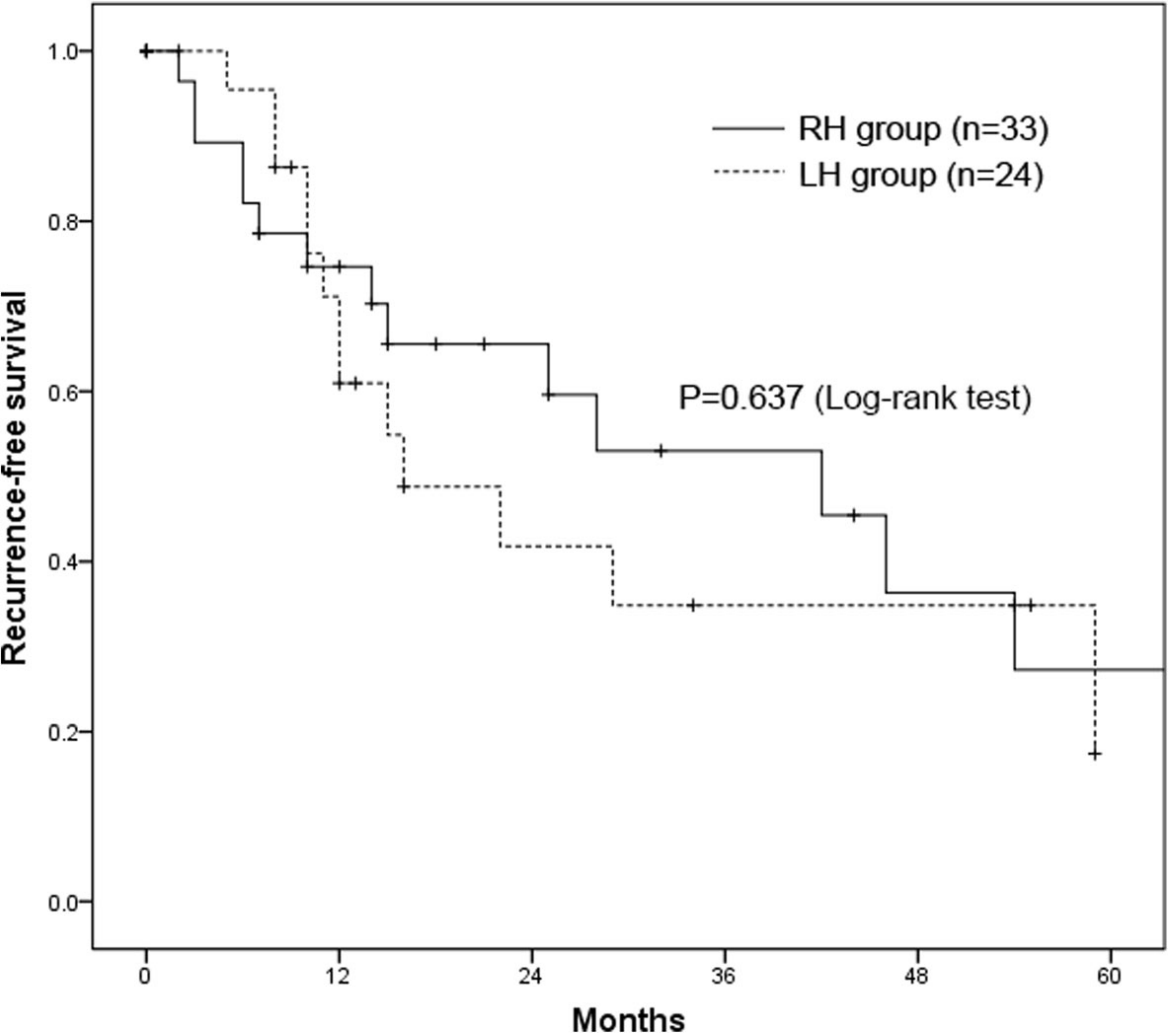
Kaplan–Meier curves showing recurrence-free survival between the RH and LH groups. RH, right-side hepatectomy; LH, left-side hepatectomy

The 1-, 3-, and 5-year OS rates for all patients were 75.2%, 49.9%, and 39.4%, respectively, and the 1-, 3-, and 5-year RFS rates were 68.3%, 43.1%, and 24.4%, respectively. The 1-, 3-, and 5-year OS rates of the RH group were 69.3%, 48.5%, and 37.7%, respectively, and those of the LH group were 82.6%, 50.6%, and 40.5% (*P =* 0.485, Fig. 2). In addition, the 1-, 3-, and 5-year RFS rates of the RH group were 76.5%, 53.8%, and 27.7%, respectively, and those of the LH group were 69.6%, 30.6%, and 15.3% (*P =* 0.637, Fig. 3).

In univariate analysis, transfusion (odds ratio, 3.48 [1.60–7.54], *P =* 0.002), PHLF (≥ grade B, 4.45 [1.83–10.82], *P =* 0.001), and N stage (≥ N1, 2.18 [1.01–4.71], *P =* 0.046) were significantly associated with OS. Multivariate analysis revealed that transfusion (3.12 [1.42–6.87], *P =* 0.005) and PHLF (≥ grade B, 4.62 [1.86–11.49], *P =* 0.001) were independent risk factors for OS (Table 2). On the other hand, transfusion (2.81 [1.30–6.05], *P =* 0.008) and LVI (3.22 [1.35–7.63], *P =* 0.008) were significantly associated with RFS; the same variables were independent risk factors in multivariate analysis (2.82 [1.28–6.20], *P =* 0.01, and 3.33 [1.34–8.23], *P =* 0.009, respectively, Table 3).

**Table 2:** Univariate and multivariate analysis of 57 patients for risk factors related to overall survival

	Univariate analysis		Multivariate analysis	
	OR (95% CI)	*P* value	OR (95% CI)	*P* value
Clinical variables				
Preoperative jaundice	0.90 (0.42–1.91)	0.784		
Preoperative biliary drainage	0.46 (0.16–1.36)	0.166		
Portal vein embolization	0.63 (0.15–2.68)	0.537		
Bismuth type IV	1.29 (0.59–2.83)	0.520		
ICG R-15 (≥ 15%)	1.46 (0.65–3.27)	0.350		
Operative variables				
Operation (LH)	0.76 (0.34–1.66)	0.491		
R1 resection	1.56 (0.68–3.58)	0.294		
Portal vein resection	0.77 (0.29–2.06)	0.615		
Transfusion	3.48 (1.60–7.54)	0.002	3.12 (1.42–6.87)	0.005
PHLF (≥ grade B)	4.45 (1.83–10.82)	0.001	4.62 (1.86–11.49)	0.001
Complication (≥ IIIA)	1.34 (0.62–2.87)	0.451		
Pathologic variables				
Differentiation (poorly)	1.38 (0.45–4.20)	0.571		
Lymphovascular invasion	2.02 (0.90–4.54)	0.087	1.17 (0.45–3.04)	0.734
T stage (≥ T3)	1.24 (0.55–2.76)	0.596		
N stage (≥ N1)	2.18 (1.01–4.71)	0.046	1.40 (0.54–3.65)	0.486

*Abbreviations*: *ICG R-15* indocyanine green retention rate at 15 minutes, *LH* left-side hepatectomy, *PHLF* post-hepatectomy liver failure, *OR* odds ratio, *CI* confidence interval

**Table 3:** Univariate and multivariate analysis of 57 patients for risk factors related to recurrence-free survival

	Univariate analysis		Multivariate analysis	
	OR (95% CI)	*P* value	OR (95% CI)	*P* value
Clinical variables				
Preoperative jaundice	1.08 (0.50–2.33)	0.829		
Preoperative biliary drainage	0.55 (0.18–1.60)	0.274		
Portal vein embolization	0.97 (0.29–3.27)	0.996		
Bismuth type IV	2.02 (0.94–4.34)	0.069	1.60 (0.73–3.48)	0.235
ICG R-15 (≥ 15%)	1.62 (0.72–3.66)	0.238		
Operative variables				
Operation (LH)	1.19 (0.56–2.55)	0.640		
R1 resection	1.17 (0.46–2.92)	0.736		
Portal vein resection	0.78 (0.31–1.96)	0.603		
Transfusion	2.81 (1.30–6.05)	0.008	2.82 (1.28–6.20)	0.01
PHLF (≥ grade B)	2.26 (0.76–6.76)	0.141		
Complication (≥ IIIA)	0.73 (0.32–1.64)	0.735		
Pathologic variables				
Differentiation (poorly)	1.76 (0.56–5.50)	0.329		
Lymphovascular invasion	3.22 (1.35–7.63)	0.008	3.33 (1.34–8.23)	0.009
T stage (≥ T3)	1.41 (0.62–3.19)	0.400		
N stage (≥ N1)	1.82 (0.82–4.04)	0.137		

*Abbreviations*: *ICG R-15* indocyanine green retention rate at 15 min, *LH* left-side hepatectomy, *PHLF* post-hepatectomy liver failure, *OR* odds ratio, *CI* confidence interval

Subgroup analysis was performed for 21 patients who had lymph node metastasis: 15 in the RH group and 6 in the LH group. Among them, 11 patients (52.4%) received adjuvant chemotherapy with gemcitabine plus cisplatin. There were no significant differences in 5-year OS and RFS rates between adjuvant and non-adjuvant treatment group (18.7% vs. 30.5%, *P* = 0.552, and 33.2% vs. 26.2%, *P* = 0.576, respectively).

## Discussion

Considering its prognostic effects on the long-term outcome, radical resection plays a major role in the treatment of hilar cholangiocarcinoma [17]. Therefore, many surgeons have made considerable efforts to adopt an aggressive surgical approach, despite technical difficulty [9, 18]. There are many considerations in determining which side of the liver to resect. The Bismuth–Corlette classification has been widely used to assess the hilar cholangiocarcinoma preoperatively [14]. It is a simple but useful method for classifying the type of tumor and deciding the surgical plan. In most cases of type IIIa or IIIb tumor, the surgical procedure is determined according to the side of the tumor. However, surgeons must choose between RH and LH for tumors extending to both sides of the bile duct to a similar level or invading hepatic inflow to the FLR. Once a surgeon decides the surgical plan, it is hard to change during surgery. Hence, clarifying the surgical outcome and long-term survival between RH and LH can be instrumental in deciding the surgical strategy for hilar cholangiocarcinoma.

Few reports have compared RH and LH in hilar cholangiocarcinoma, and the impact of the side of the liver resection has not yet been established [12, 13]. It could be considered that RH has an advantage over LH for achieving R0 resection. The tumor tends to invade the right hepatic artery or portal vein because biliary confluence leans to the right side of the vascular confluence [9, 11, 19]. This could lead surgeons performing LH to choose whether to stop further resection or to conduct aggressive vascular reconstruction. Various studies have demonstrated a high incidence of vascular invasion leading to reconstruction in LH [20, 21]. Nagino et al. reported acceptable mortality and better long-term survival rates following major hepatectomy with simultaneous vascular reconstruction for hilar cholangiocarcinoma consisting of predominantly LH [10]. However, although combined portal vein resection and reconstruction are considered to be a certain option to increase resectability with acceptable morbidity [22], hepatic artery reconstruction could still be technically difficult and cause serious complications.

Furthermore, achieving a negative proximal ductal margin is another reason that makes R0 resection difficult. Some authors have asserted that a negative proximal ductal margin can be more easily achieved in RH because the left extrahepatic bile duct to the bifurcation is longer than that of the right liver and there is less variation in the segmental anatomy of the left liver [23]. However, estimation of longitudinal tumor extent along the bile ducts should be performed before determination of side of liver resection and there was no difference in the proportion of R0 resections (approximately 75%) between groups in the present study which was comparable to those reported previously [8, 24, 25]. In this study, the extent of tumor was the most important consideration in determining the side of liver resection. Then, the tumor invasion of portal vein or hepatic artery and possibility of reconstruction were evaluated with various imaging studies. However, in case in which R0 resection was possible only with one of both sides and vascular invasion to FLR was reconstructible, the side of resection was determined depending on the tumor extent. In case that either side of resection can be considered to achieve a R0 resection, we do not recommend right-side resection with concerns of PHLF based on our results. In terms of achieving a negative proximal ductal margin, we did not routinely perform a frozen biopsy because the proximal bile duct was resected at the highest achievable level [26]. As a result, we reconstructed each segmental bile duct with more than three openings in almost all cases. Therefore, the authors assume that achieving R0 resection depends more on expertise to acquire and reconstruct the proximal bile duct margin as high level as possible, despite several anatomical issues.

Surgical resection for hilar cholangiocarcinoma has higher morbidity and mortality than those of any other operation in hepatobiliary pancreatic surgery [27]. A previous study reported that postoperative complications, including PHLF, occur more frequently in RH than LH [28]. In this study, a postoperative complication rate above Clavien–Dindo grade IIIA was reported in about 40% of both groups, while the hospital stay and 90-day mortality rates did not differ between the groups. PHLF tended to occur more frequently in the RH group than in the LH group (21.2% vs. 8.3%) but the difference was not statistically significant. A small FLR volume associated with severe PHLF is one of the essential considerations in planning surgical strategy [29, 30]. We focused on two ways to prevent PHLF in RH for hilar cholangiocarcinoma, namely, the aggressive use of preoperative biliary drainage for FLR, and PVE. Resection of the jaundiced liver could lead to severe morbidity and mortality [31, 32]. Although there is controversy regarding preoperative biliary drainage, it could relieve preoperative cholangitis and prevent PHLF by resolving obstructive jaundice [33, 34].

Furthermore, maximizing FLR by PVE has allowed better postoperative recovery and reduced the occurrence of PHLF [35]. In this study, six patients (18.2%) in the RH group underwent PVE, with the criteria of performing PVE for FLR of less than 35% as assessed through CT volumetry. A subgroup analysis of the RH group showed no differences in the occurrence of PHLF (≥ grade B) between the PVE and non-PVE groups, suggesting the preventive effect of PVE for the occurrence of PHLF. Although multivariate analysis revealed that PVE was not a significant risk factor for survival, it should be encouraged for patients who are likely to develop PHLF considering that PHLF was an independent risk factor for overall survival. Some groups use PVE more actively, with the criteria of performing right hemihepatectomy or FLR of less than 40% [35]. However, it could be excessive criteria considering a relatively well-preserved liver function in patients with hilar cholangiocarcinoma and a similar proportion of the occurrence of PHLF in both PVE and non-PVE groups in this study. Besides, this invasive procedure has a disadvantage of delaying operation by several weeks for patients without jaundice. Therefore, PVE should be performed on selected patients, considering the underlying liver function and extent of liver resection.

The 5-year OS and RFS were 39.4% and 24.4%, respectively, and no significant difference was observed in OS and RFS between the RH and LH groups. This finding may be due to the similar proportions of R0 resection and pathologic characteristics in both groups. As described above, there were several differences in anatomy and extent of liver resection between RH and LH. However, there were no differences in long-term outcomes following radical resection between groups with similar invasiveness. Multivariate analysis revealed that transfusion was a common risk factor for OS and RFS. Aside from technical aspects, it could be inferred that patients receiving transfusion had worse underlying liver function or aggressive tumor characteristics. It has been reported that transfusion negatively affects not only perioperative outcomes with poor immune modulation but also cancer-related mortality [36]. Although extensive resection is mandatory for the surgical resection of hilar cholangiocarcinoma, we should strive to reduce unnecessary transfusion and optimize patient condition preoperatively. Furthermore, subgroup analysis for patients who had lymph node metastasis revealed that there were no significant differences in long-term outcomes between adjuvant and non-adjuvant treatment group. Although this result could imply the superiority of surgical resection as curative treatment and the limited role of adjuvant chemotherapy, it is hard to conclude owing to the small sample size of each group.

A limitation of this study was its retrospective design with relatively small sample size. Although hilar cholangiocarcinoma accounts for 60–70% of extrahepatic cholangiocarcinoma, the number of cases in a single center is limited. We hope that future multi-center studies involving larger sample sizes will produce more concrete results.

## Conclusions

This study suggests that the side of the liver resection did not impact perioperative and long-term outcomes in patients undergoing curative-intent resection for hilar cholangiocarcinoma. We recommend planning a surgical strategy based on the possibility of achieving radical resection with efforts to reduce morbidity and mortality considering the anatomical differences between RH and LH.

## Data Availability

All data generated or analyzed during this study are included in this published article.

## Abbreviations

CT: Computed tomography
ENBD: Endoscopic nasobiliary drainage
ERCP: Endoscopic retrograde cholangiopancreatography
FLR: Future liver remnant
ICG: Indocyanine green
LH: Left-side hepatectomy
MR: Magnetic resonance
OS: Overall survival
PET: Positron emission tomography
PHLF: Post-hepatectomy liver failure
PTBD: Percutaneous transhepatic biliary drainage
PVE: Portal vein embolization
RFS: Recurrence-free survival
RH: Right-side hepatectomy
